# Absolute signal of stimulated Raman scattering microscopy: A quantum electrodynamics treatment

**DOI:** 10.1126/sciadv.adm8424

**Published:** 2024-12-11

**Authors:** Wei Min, Xin Gao

**Affiliations:** Department of Chemistry, Columbia University, New York, NY 10027, USA.

## Abstract

The advent of stimulated Raman scattering (SRS) microscopy has launched a rapidly growing field in chemical imaging with broad impact. Although the physical picture seems to be well understood from classical models, prediction of absolute SRS signals remains a challenge. Here, we present a quantum electrodynamics treatment of the newly introduced stimulated Raman cross section. The resulting formula for calculating the absolute SRS signal is simple and differs from the commonly used relations by only one factor. We demonstrate the utility of this formula in a broad range of crucial applications of SRS microscopy, including stimulated Raman enhancement factor (>10^8^ times), signal-to-noise ratio (SNR) of typical imaging experiments, population saturation under high power excitation, and energy deposition during stimulated Raman photothermal microscopy. In particular, the theory predicts that SRS microscopy is almost always more sensitive than spontaneous Raman microscopy for chemical imaging.

## Teaser

A formula to calculate the absolute stimulated Raman scattering (SRS) signal is derived from quantum electrodynamics (QED) theory.

## INTRODUCTION

Stimulated Raman scattering (SRS) microscopy has gradually matured into a powerful and popular chemical imaging technique, affecting various fields of research including biological imaging, analytical chemistry, chemical biology, and material science (*1*). Since its first invention, the underlying physics has often been formulated by a classical model in the literature of SRS microscopy (*2*–*9*). In brief, the incident light fields (*E*_p_ and *E*_S_) induce polarizations at the same frequencies of the incoming pump and Stokes beams

$$P\left(\omega_{p}\right)=6\varepsilon_{0}\chi_{R}^{\left(3\right)}\left(\Omega \right)\;E_{p}\;{|E_{S}|}^{2}$$ (1a)

$$P\left(\omega_{S}\right)=6\varepsilon_{0}\chi_{R}^{\left(3\right)}\left(\Omega \right)\;E_{S}\;{|E_{p}|}^{2}$$ (1b)

where ε_0_ is the vacuum permittivity and $\chi_{R}^{\left(3\right)}$ is the Raman-dependent third-order nonlinear susceptibility of the material. Consequently, as *P*(ω_p_) and *P*(ω_S_) radiate as the source term in the Maxwell equation, they produce the nonlinear field components, *E*_SRG_ and *E*_SRL_, at the far-field detector. Having the same frequencies as the incoming beams and being coherent, *E*_SRG_ and *E*_SRL_ interfere with the incident field components. The interference produces stimulated Raman loss (SRL) of the pump beam and stimulated Raman gain (SRG) of the Stokes beam

$$∆I_{\text{SRL},\text{SRG}}\propto \mathrm{Ιm}\left\{\chi^{\left(3\right)}\right\}\cdot {|E_{p}|}^{2}\;{|E_{S}|}^{2}$$ (2)

where the imaginary part describes the dissipative response of χ^(3)^. The square of *E*_p_ and *E*_S_ scales with the intensity, *I*, of the incident beams. Thus, the following form is also commonly used

$${\Delta I}_{\text{SRL},\text{SRG}}\propto \text{Im}\left\{\chi^{\left(3\right)}\left(\Omega \right)\right\}\cdot I_{p}\cdot I_{S}$$ (3)

Despite its conceptual simplicity, the numerical values of Im{χ^(3)^} are rarely reported for real molecular systems, and the full quantum mechanical calculation is an expensive and nontrivial task. Fortunately, Im{χ^(3)^} has been theoretically shown to be linear with respect to molecular concentration and the Raman cross section σ_Raman_, which has been measured and documented for many molecules over the decades. Then, Eq. 3 converts to a more widely quoted form as given in the literature (*2*, *5*, *10*–*12*)

$$∆I_{\text{SRL},\text{SRG}}\propto [c]\cdot \sigma_{\text{Raman}}\cdot I_{\text{pump}}\cdot I_{\text{Stokes}}$$ (4)

Equation 4 correctly and readily explains the experimental observations of the SRS process including the linear laser intensity dependence on *I*_pump_ and *I*_Stokes_, the linear dependence on analyte concentration, the spectral correspondence with regular Raman spectrum, and the energy transfer nature between laser fields and molecules. As such, it has been the key theoretical basis summarized in major reviews of SRS microscopy for the past decade (*2*–*12*).

Equation 4, however, is merely a proportionality. The units of both sides obviously do not match each other. Researchers can only use it to make relative comparisons within the same experimental setting by using proper references. It becomes less useful in understanding the SRS signal in the absolute sense. It cannot predict the absolute voltage at the photodetector or the amount of energy deposition during each excitation pulse. Without such information, it is also difficult to answer questions like how much signal to noise one can obtain during imaging experiments, and whether vibrational excitation has approached saturation. For instance, an experimental estimation that SRS microscopy can accelerate the vibrational transition by >10^7^ times compared to regular Raman was reported^2^, but has remained unexplained thus far. To answer all these questions, a true equality is required. We are not aware of a popular formula that SRS microscopists use to predict the absolute SRS signal.

Recently, stimulated Raman cross section, σ_SRS_, was introduced to characterize intrinsic molecular response during SRS (*13*). It was proposed after making an analogy to two-photon absorption cross section (which has a dimension of cm^4^ s, named after its developer Göppert-Mayer)

$$R_{\text{SRS}}=N\cdot \sigma_{\text{SRS}}\cdot \phi_{\text{pump}}\cdot \phi_{\text{Stokes}}$$ (5)

*N* is the number of bonds corresponding to the measured vibrational mode, and ϕ_pump_ and ϕ_Stokes_ are the photon flux (photon cm^−2^ s^−1^) of the pump and Stokes beam, respectively. The measurement results of σ_SRS_ have revealed surprising insights. Different from the prevailing view that σ_Raman_ is always many orders of magnitude (up to 10^14^) smaller than its electronic absorption counterpart, σ_SRS_ can even be much larger than the two-photon absorption cross section of similar molecules (*13*). This initial treatment is phenomenological in that σ_SRS_ had to be measured experimentally. More recently, we are inspired by Einstein’s A and B coefficients for spontaneous and stimulated emission, and derived an analogous equation for Raman scattering (*14*). The resulting equation connects the regular Raman cross sections with stimulated Raman cross sections through a factor arising from an effective vacuum electromagnetic field (i.e., vacuum fluctuation). A physical constraint was adopted there without explicitly referring to full quantum mechanics, and consequently, the analytic expression of σ_SRS_ was not given (*14*).

Here, we provide a quantum electrodynamics (QED) treatment for σ_SRS_ in which both the matter and the electromagnetic field are quantized. Equation 16 gives the analytic expression of σ_SRS_ from first principles. The numerical estimation of σ_SRS_ agrees with the experimental measurements of model compounds. The comparison between σ_SRS_ and σ_Raman_ reproduces the earlier equation between these two Raman cross sections. This bridge naturally leads to a simple formula, Eq. 30, for the absolute SRS signal by using the well-documented σ_Raman_. Compared to Eq. 4, the newly introduced formula incorporates one additional factor, which we interpret as the effective, molecule-dependent, vacuum field intensity. We further apply this formula to quantitatively address a range of important chemical imaging issues. Equipped with predictive and explanatory power, this set of formulas shall provide an instructive framework to facilitate quantitative applications of SRS in the coming ages.

## RESULTS

### QED derivation of stimulated Raman cross section

Here, we derive σ_SRS_ using QED theory in which both fields and matter are quantum mechanical (*15*–*22*). We start with Fermi’s golden rule in the delta function representation

$$r\left(E\right)=\frac{2\pi }{\hbar }{|M_{\mathrm{fi}}|}^{2}\delta \left(E_{f}-E_{i}\right)$$ (6a)

where *r* is the transition probability and *M_fi_* is the matrix element of the coupling between the initial state *i* to the final state *f*, and the Dirac delta function imposes conservation of energy. Equation 6a can also be expressed in angular frequency instead of energy

$$r\left(\Omega_{v}\right)=\frac{2\pi }{\hbar^{2}}{|M_{\mathrm{fi}}|}^{2}\delta \left(\omega_{S}+\Omega_{v}-\omega_{p}\right)$$ (6b)

where we use Ω_v_ to denote the molecular intrinsic frequency of Raman mode. The energy conservation is ensured by the frequency restriction between the incident pump and Stokes laser beams and the frequency of the excited Raman mode. In other words, only vibrational state satisfying ω_S_ + Ω_v_ − ω_p_ = 0 can be reached. Note that the transition probability *r* has a unit of s^−1^, thus representing the probability of transition per unit time (i.e., rate). Here, we have also assumed that the pump and Stokes beams are chirp-free and spatiotemporally overlapped, as is typically true in standard picosecond SRS experiments.

From a QED perspective, this interaction results in a transition of the radiation field from an initial state of |*n*_p_(***k***_**p**_), *n*_S_(***k***_**S**_)〉 to a final state of |(*n*_p_ − 1)(***k***_**p**_), (*n*_S_ + 1)(***k***_**S**_)〉, where *n*_p_ and *n*_S_ are the number of photons in the pump beam mode and the Stokes beam mode, respectively, and ***k***_**p**_ and ***k***_**S**_ are the wave vector (note that the polarization information is also included to keep the notation simpler) corresponding to frequency ω_p_ and ω_S_, respectively. In general, second-order perturbation theory can calculate the transition matrix element as

$$M_{\mathrm{fi}}=\sum_{m}\frac{\left\langle f|{\hat{H}}_{\text{ED}}|m\right\rangle \left\langle m|{\hat{H}}_{\text{ED}}|i\right\rangle }{E_{i}-E_{m}}$$ (7)

where ${\hat{H}}_{\text{ED}}$ is the electric dipole interaction Hamiltonian, which, in the long wavelength approximation, keeps the dominant contribution from the expansion of the electric potential energy and neglects the magnetic and the high-order nonlinear terms (*17*). The *m* summation runs over all the intermediate virtual states, and the energy E_m_ in the denominator includes contributions from both the molecule and the field.

${\hat{H}}_{\text{ED}}$ takes a form as ${\hat{H}}_{\text{ED}}=-\hat{\mu }\cdot {\hat{E}}_{T}$, where $\hat{\mu }$ is the dipole moment operator and ${\hat{E}}_{T}$ is the operator of the transverse electric field

$${\hat{E}}_{T}\left(r\right)=i\sum_{k}\sqrt{\frac{\hbar \omega_{k}}{2\varepsilon_{0}V}}\cdot e_{k}\cdot \left({\hat{a}}_{k}e^{ik\cdot r}-{\hat{a}}_{k}^{†}e^{-ik\cdot r}\right)$$ (8)

where ${\hat{a}}_{k}$ and ${\hat{a}}_{k}^{†}$ are the annihilation and creation operators, respectively, and ***e*** designates the unit polarization vector that points to the polarization direction of the fields. They exhibit the property of destroying or creating a quantum of energy, or a photon in QED, as manifested by the simple structures of the only nonvanishing matrix elements: $\left\langle n-1|\hat{a}|n\right\rangle =\sqrt{n}$ and $\left\langle n+1|{\hat{a}}^{†}|n\right\rangle =\sqrt{n+1}$. The interaction Hamiltonian can take both the $\hat{\mu }\cdot {\hat{E}}_{T}$ form adopted here and the other $\hat{p}\cdot \hat{A}$ form involving the operator of the vector potential $\hat{A}$, which produces the same results. The full quantum ${\hat{H}}_{\text{ED}}=-\hat{\mu }\cdot {\hat{E}}_{T}$ is analogous to the semiclassical case of V=−μ·E used in classical treatment.

**Fig. 1.**
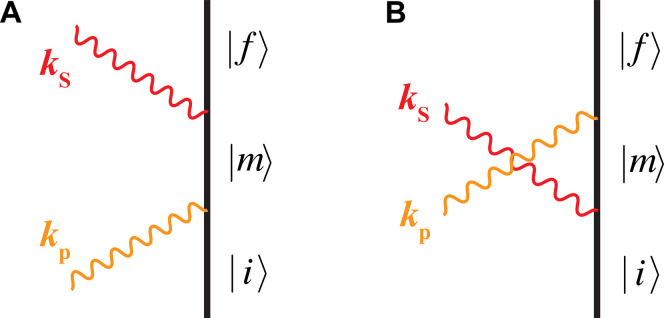
Feynman diagram of SRS. (**A**) Direct pathway where a molecule initially in the ground state ∣i⟩ absorbs a pump photon with wave vector ***k***_p_ and transitions to a virtual state ∣m⟩. Subsequently, the molecule emits a Stokes photon ***k***_S_ and transitions to the final state ∣f⟩. (**B**) Inverse process where the molecule first emits a Stokes photon before absorbing the pump photon. Both processes contribute to the net Raman scattering effect.

As depicted in Fig. 1, there are two time-ordered pathways that can contribute to the matrix elements in the SRS process. The first is where a molecule is transitioning from the initial state *i* to a virtual intermediate state *m* with the incident photon destroyed from the pump beam, followed by the emission of a new photon to the Stokes beam and molecular transition to the final state *f*. The second is similar, with the photon creation “preceding” the photon destruction. All these formulas and considerations above allow explicit evaluation of the matrix element in Eq. 7 as

$$M_{\mathrm{fi}}=\sqrt{\frac{n_{p}\hbar \omega_{p}}{2\varepsilon_{0}V}}\sqrt{\frac{\left(n_{S}+1\right)\hbar \omega_{S}}{2\varepsilon_{0}V}}\left[\sum_{m}\left\{\frac{\left(\mu^{\text{fm}}\cdot e_{S}\right)\left(\mu^{\text{mi}}\cdot e_{p}\right)}{E_{m}-E_{i}-\hbar \omega_{p}-\mathrm{i\hbar}\gamma }+\frac{\left(\mu^{\text{fm}}\cdot e_{p}\right)\left(\mu^{\text{mi}}\cdot e_{S}\right)}{E_{m}-E_{i}+\hbar \omega_{S}-i\hbar \gamma }\right\}\right]$$ (9a)

where **μ** is the dipole moment vector. The first term in the summation with energy difference in the denominator corresponds to diagram A, and the second term with energy sum in the denominator corresponds to diagram B. iℏγ is included as a damping term. Clearly, $\sqrt{n_{p}}$ arises as a consequence of ${\hat{a}}_{k}$ acting on the transition from |*n*_p_(***k***_**p**_)〉 to |(*n*_p_ − 1)(***k***_**p**_)〉; $\sqrt{n_{S}+1}$ arises as a consequence of ${\hat{a}}_{k}^{†}$ acting on the transition from |*n*_S_(***k***_**S**_)〉 to |(*n*_S_ + 1)(***k***_**S**_)〉. For simplicity, we use M to denote the summation part

$$M\equiv \sum_{m}\left\{\frac{\left(\mu^{\text{fm}}\cdot e_{S}\right)\left(\mu^{\text{mi}}\cdot e_{p}\right)}{E_{m}-E_{i}-\hbar \omega_{p}-\mathrm{i\hbar \gamma}}+\frac{\left(\mu^{\text{fm}}\cdot e_{p}\right)\left(\mu^{\text{mi}}\cdot e_{S}\right)}{E_{m}-E_{i}+\hbar \omega_{S}-i\hbar \gamma }\right\}$$ (9b)

Substituting the matrix element to Eq. 6b yields the rate of SRS

$$r_{\text{SRS}}\left(\Omega_{v}\right)=\frac{\pi \omega_{p}\omega_{S}}{2\varepsilon_{0}^{2}c^{2}}\cdot \left(\frac{n_{p}c}{V}\right)\left[\frac{\left(n_{S}+1\right)c}{V}\right]\cdot |M{|}^{2}\cdot \delta \left(\omega_{S}+\Omega_{v}-\omega_{p}\right)$$ (10)

In realistic situations, the intrinsic Raman mode of the molecule does not have a perfectly well-defined transition frequency but is always spread into a continuous distribution by various broadening mechanisms. One often expresses this effect by stating that the final state is spread into a density of final state continuum (*22*). In the context of Raman scattering, this density of states is essentially the normalized line shape profile *G*(Ω_v_): $\int_{0}^{\infty }G\left(\Omega_{v}\right)\cdot d\Omega_{v}=1$. Then, the final rate must be averaged over all possible values of the transition frequency, i.e., via integration over the line shape profile.

$$r_{\text{SRS}}\left(\Omega_{v}\right)=\frac{\pi \omega_{p}\omega_{S}}{2\varepsilon_{0}^{2}c^{2}}\cdot \left(\frac{n_{p}c}{V}\right)\left[\frac{\left(n_{S}+1\right)c}{V}\right]\cdot {|M|}^{2}\cdot \int_{0}^{\infty }G\left(\Omega_{v}\right)\cdot \delta \left(\omega_{S}+\Omega_{v}-\omega_{p}\right)\cdot d\Omega_{v}$$ (11)

Then, one has

$$r_{\text{SRS}}\left(\Omega_{v}\right)=\frac{\pi \omega_{p}\omega_{S}}{2\varepsilon_{0}^{2}c^{2}}\cdot \left(\frac{n_{p}c}{V}\right)\left[\frac{\left(n_{S}+1\right)c}{V}\right]\cdot |M{|}^{2}\cdot G\left(\Omega_{v}=\omega_{p}-\omega_{S}\right)$$ (12)

where the notation *G*(Ω_v_ = ω_p_ − ω_S_) means that the line shape profile is to be evaluated at the frequency difference, ω_p_ − ω_S_, of the incident pump and Stokes laser beams. Note that this notation applies to any frequency difference of the incident beams, provided the line shape function is known either experimentally or through a phenomenological function such as a Lorentzian profile.

The incident photon flux ϕ is defined as the number of photons per unit time per unit area that crosses a given point. If the volume *V* consists of a photon beam of area *A* and length *L*, then in a time *t* = *L*/*c* the number of the photons crossing is simply the number of photons, *n*, within this volume. Here, we have assumed the refractive index to be 1. Thus one can verify that

$$\phi =\frac{n}{A\cdot t}=\frac{n}{A\cdot \left(L/c\right)}=\frac{n\cdot c}{V}$$ (13)

Obviously, *n*_S_ + 1 ≈ *n*_S_ for strong laser beams used in SRS microscopy experiments. Introducing the photon flux to Eq. 12 yields

$$r_{\text{SRS}}\left(\Omega_{v}\right)=\frac{\pi \omega_{p}\omega_{S}}{2\varepsilon_{0}^{2}c^{2}}\cdot \phi_{\text{pump}}\cdot \phi_{\text{Stokes}}\cdot {|M|}^{2}\cdot G\left(\Omega_{v}=\omega_{p}-\omega_{S}\right)$$ (14)

In most experiments with gases, liquids, and biomaterials, Raman modes are randomly orientated. Hence, an additional factor of ^1^/_9_ is added to compensate for the dipole-field alignment of the randomly oriented molecules. This arises from the second-order light-molecule interaction, as each interaction contributes to a factor of ^1^/_3_ as in the case of linear interaction. Subsequently, we have

$$r_{\text{SRS}}\left(\Omega_{v}\right)=\frac{\pi \omega_{p}\omega_{S}}{18\varepsilon_{0}^{2}c^{2}}\cdot \phi_{\text{pump}}\cdot \phi_{\text{Stokes}}\cdot {|M|}^{2}\cdot G\left(\Omega_{v}=\omega_{p}-\omega_{S}\right)$$ (15)

Comparing the rate equation Eq. 15 to the definition of Eq. 5, we arrive at the expression for stimulated Raman cross section

$$\sigma_{\text{SRS}}\left(\Omega_{v}\right)=\frac{\pi \omega_{p}\omega_{S}}{18\varepsilon_{0}^{2}c^{2}}\cdot {|M|}^{2}\cdot G\left(\Omega_{v}=\omega_{p}-\omega_{S}\right)$$ (16a)

To our knowledge, this is the first time an expression is formally given for σ_SRS_.

The line shape function *G*(Ω_v_) of Raman spectral peaks can often be modeled with a Lorentzian profile $L\left(\tilde{\nu }\right)=\frac{1}{\pi }\frac{\frac{1}{2}\Gamma }{{\left(\tilde{\nu }-{\tilde{\nu }}_{0}\right)}^{2}+{\left(\frac{1}{2}\Gamma \right)}^{2}}$, with Γ being its full width at half maximum (FWHM). Typically, in an SRS experiment, the pump and Stokes beams are tuned to match the peak position, Ω_0_, of the Raman mode, i.e., ω_p_ − ω_S_ = Ω_0_. *G*(Ω_v_ = Ω_0_) evaluated at the peak of the Lorentzian profile is $L\left(\tilde{\nu }\right){|}_{\tilde{\nu }={\tilde{\nu }}_{0}}=\frac{2}{\pi \Gamma }$. At this peak position,

$$\sigma_{\text{SRS}}\left(\Omega_{v}=\Omega_{0}\right)=\frac{\omega_{p}\omega_{S}}{9\varepsilon_{0}^{2}c^{2}\Gamma }\cdot {|M|}^{2}$$ (16b)

Numerical evaluations of Eq. 16 for model compounds, both the off-resonance case and the electronic resonance case, are finding good agreement with experimental measurement (details in note S1). Hence, the intrinsically strong σ_SRS_, initially observed experimentally, can now be explained from first principles.

### QED treatment of spontaneous Raman cross section

Quantum mechanical expression for σ_Raman_ has been given in the literature (*16*–*18*). Yet, it will be constructive to derive it, as some intermediate steps will be needed for subsequent analysis. QED considers the coupling with vacuum state in a straightforward manner by assigning *n*_S_ = 0 in the matrix element of Eq. 9. In spontaneous Raman, all vacuum modes are accessible for the scattered photon, and thus, we need to take the sum of different modes with different wave vectors ***k***. Repeating the steps leading to Eq. 10, assigning *n*_S_ = 0 and taking the sum of different modes result in

$$r_{\text{Raman}}=\sum_{k_{S}}\frac{\pi \omega_{p}\omega_{S}}{2\varepsilon_{0}^{2}c^{2}}\cdot \left(\frac{n_{p}c}{V}\right)\left(\frac{c}{V}\right)\cdot {|M|}^{2}\cdot \delta \left(\omega_{S}+\Omega_{v}-\omega_{p}\right)$$ (17)

The summation over all the scattered wave vectors can be converted into an integration over frequency ω_S_ and solid angle Ω, a common practice used in electrodynamic theory (*17*)

$$\sum_{k_{S}}\to \frac{V}{{\left(2\pi \right)}^{3}}\iint d\omega_{S}d\Omega \frac{\omega_{S}^{2}}{c^{3}}$$ (18)

Then, the rate in Eq. 17, now considered as a differential rate into the solid angle dΩ, becomes

$$\frac{dr_{\text{Raman}}}{\mathrm{d\Omega}}=\frac{1}{16\pi^{2}\varepsilon_{0}^{2}c^{4}}\cdot \int \omega_{p}\omega_{S}^{3}\cdot \phi_{p}\cdot {|M|}^{2}\cdot \delta \left(\omega_{S}+\Omega_{v}-\omega_{p}\right)\cdot d\omega_{S}$$ (19)

A nonzero contribution to the total rate would require Raman resonance conditions that satisfy ω_S_ = ω_p_ − Ω_v_. The frequency integration is readily performed with the use of the delta function

$$\frac{dr_{\text{Raman}}}{\mathrm{d\Omega}}=\frac{1}{16\pi^{2}\varepsilon_{0}^{2}c^{4}}\cdot \omega_{p}\omega_{S}^{3}\cdot \phi_{p}\cdot {|M|}^{2}$$ (20)

Integrating dΩ over all the spatial angle (4π) and assuming isotropic scattering under two polarizations of light, and also adding the ^1^/_9_ factor for orientation, then the total Raman scattering rate can be evaluated at

$$r_{\text{Raman}}=\frac{1}{18\pi \varepsilon_{0}^{2}c^{4}}\cdot \omega_{p}\omega_{S}^{3}\cdot \phi_{p}\cdot {|M|}^{2}$$ (21)

The isotropic assumption (i.e., depolarization ratio ρ = 1) is a common practice in Raman literature (*16*, *18*, *19*, *21*). The actual values for ρ depend on the group symmetry and resonance conditions of the vibrational mode, and Eq. 21 can be modified accordingly.

If defined as $P_{\text{Raman}}=\sigma_{\text{Raman}}\cdot I_{p}$, where *P* is the power scattered into the Stokes channel, then we arrive at

$$\sigma_{\text{Raman}}=\frac{\omega_{S}^{4}}{18\pi \varepsilon_{0}^{2}c^{4}}\cdot {|M|}^{2}$$ (22)

### A simple formula for absolute SRS signal

The explicit expressions of stimulated Raman and spontaneous Raman cross sections above allow us to investigate their connection. As shown in Eq. 16a, σ_SRS_ itself is a function of Ω_v_. Then, one can verify the following integral form regarding σ_Raman_ in Eq. 22

$$\sigma_{\text{Raman}}=\frac{\omega_{S}^{3}}{\pi^{2}c^{2}\omega_{p}}\int \sigma_{\text{SRS}}\left(\Omega_{v}\right)d\Omega_{v}$$ (23)

This explicitly states that, apart from a factor, Raman cross section is an integration of the stimulated Raman cross section. Theoretically, the concept of “Raman line shape” is not needed in modeling spontaneous Raman scattering, both quantum mechanically and pure classically. In contrast, both classical and quantum theories have to incorporate a vibrational damping term (Raman line broadening) to explain SRS; otherwise, the energy flow from the fields to the molecules would become unbounded (i.e., infinity).

As most SRS experiments tune the pump and Stokes beams to match the peak position, Ω_0_, of the Raman mode, we can find the connection between the peak value of σ_SRS_(Ω_v_ = Ω_0_) and σ_Raman_ by comparing Eq. 16b with Eq. 22 as

$$\sigma_{\text{Raman}}=\frac{\omega_{S}^{3}\Gamma }{2\pi c^{2}\omega_{p}}\sigma_{\text{SRS}}\left(\Omega_{v}=\Omega_{0}\right)$$ (24)

This reproduces the exact equation that was recently derived using the concept of virtual vacuum photons but without explicitly referring to full quantum mechanics (*14*). Now, we have proved its validity in an independent and more straightforward way. The factor in front of σ_SRS_($\Omega_{v}=\Omega_{0}$) together displays a unit of photon/(s cm^2^), providing a physical meaning of an effective photon flux arising from the vacuum fluctuation

$$\sigma_{\text{Raman}}=\phi_{\text{vacuum}}\cdot \sigma_{\text{SRS}}\left(\Omega_{v}=\Omega_{0}\right),\text{where}\;\phi_{\text{vacuum}}=\frac{\omega_{S}^{3}\Gamma }{2\pi c^{2}\omega_{p}}$$ (25)

This bridge equation teaches us that, while σ_SRS_ characterizes the intrinsic, vacuum-decoupled, molecular response during Raman scattering and turns out to be not weak after all, the extremely feeble ϕ_vacuum_ makes σ_Raman_ appear small (*14*). Hence, a picture of Raman duality is emerging. This relation carries the same spirit as Einstein’s coefficients connecting spontaneous emission and stimulated emission.

Now, we are in a position to derive a formula that experimentalists can readily use for predicting absolute SRS signals. With the establishment of bridge Eq. 25, the rate of SRS in Eq. 15 can be recast into a form involving σ_Raman_

$$R_{\text{SRS}}\left(\Omega_{v}=\Omega_{0}\right)=N\cdot \left(\frac{\sigma_{\text{Raman}}}{\phi_{\text{vacuum}}}\right)\cdot \phi_{\text{pump}}\cdot \phi_{\text{Stokes}}$$ (26)

The SRS signal is often expressed as an energy flux (i.e., power scattered into the Stokes channel). Multiplying ℏω_S,_ the energy of the Stokes photons, on both sides of Eq. 26, one gets *P*_SRG_, the energy flux (J s^−1^) of SRG. We then also multiply ℏω_p_ to both ϕ_pump_ and ϕ_vacuum_ in Eq. 26, and we arrive at

$$P_{\text{SRG}}\left(\Omega_{v}=\Omega_{0}\right)=N\cdot \left(\frac{\sigma_{\text{Raman}}}{\phi_{\text{vacuum}}\cdot {\hbar \omega }_{p}}\right)\cdot I_{\text{pump}}\cdot I_{\text{Stokes}}$$ (27)

Using Eq. 25 again, we can evaluate the term

$$\phi_{\text{vacuum}}\cdot {\hbar \omega }_{p}=\frac{{\hbar \omega }_{S}^{3}\Gamma }{2\pi c^{2}}\equiv I_{\text{vacuum}}$$ (28)

This term clearly carries a unit of light intensity [in J/(s cm^2^)]. As ϕ_vacuum_ was defined previously, we are prompted to define this term as an effective virtual photon intensity, *I*_vacuum_, arising from the vacuum fluctuation. It is illuminating to explore the connection between *I*_vacuum_ and Einstein’s coefficients derived in the context of blackbody radiation: $A_{21}=\frac{{\hbar \omega }_{21}^{3}}{\pi^{2}c^{3}}B_{21}$ (*23*). It is meaningful to express *I*_vacuum_ as

$$I_{\text{vacuum}}=\left(\frac{A_{21}}{B_{21}}\right)\cdot \left(\frac{\pi \Gamma }{2}\right)\cdot c$$ (29)

Recognizing that $\frac{\pi \Gamma }{2}$ denotes the Raman peak width, we suggest *I*_vacuum_ be understood as being related to a Raman analog of Einstein’s coefficients.

Finally, we arrive at

$$P_{\text{SRG}}\left(\Omega_{v}=\Omega_{0}\right)=N\cdot \left(\frac{\sigma_{\text{Raman}}}{I_{\text{vacuum}}}\right)\cdot I_{\text{pump}}\cdot I_{\text{Stokes}}$$ (30)

which is a key result of this work. It not only reproduces all the previous dependence on experimental parameters but also gives an absolute value. The same rate equation for SRL can also be derived for the pump beam (note S2). Compared to Eq. 4, we have figured out the missing factor, *I*_vacuum_, that would be needed to allow for a complete equality. Given that σ_Raman_ has been measured and documented for many molecules over the decades, we expect Eq. 30 to have practical utility for experimentalists. In the following part, we shall apply this formula to a range of microscopy issues.

### Stimulated Raman enhancement factor over 10^8^ times

An enhancement factor (EF) of about 10^7^ to 10^8^ is measured in previous literature in an attempt to quantify the extent of rate acceleration of vibrational transition in SRS versus spontaneous Raman (*2*, *13*, *24*). This is estimated by experimental measurements on model compounds without a solid theoretical foundation. Equipped with the newly introduced formula, we can theoretically estimate the EF by taking the ratio between the SRS signal and the spontaneous Raman signal as

$$\text{EF}=\frac{P_{\text{SRG}}}{P_{\text{Raman}}}=\frac{N\cdot \left(\frac{\sigma_{\text{Raman}}}{I_{\text{vacuum}}}\right)\cdot I_{\text{pump}}\cdot I_{\text{Stokes}}}{N\cdot \sigma_{\text{Raman}}\cdot I_{p}}=\frac{I_{\text{Stokes}}}{I_{\text{vacuum}}}$$ (31)

where we have assumed identical parameters for the pump beam for the sake of simplicity. This is justified by realizing that the result of the spontaneous Raman signal collected over an extended time duration remains unchanged when switching the pump laser from a continuous wave mode to a pulsed mode while keeping the average laser power constant. Equation 31 supports our picture that SRS and spontaneous Raman can both be thought of as stimulated events and their difference lies in the strength of the Stokes beams: *I*_Stokes_ captures the external light source, and *I*_vacuum_ can be regarded as a collective behavior of all the virtual photons from vacuum.

Let us evaluate a model compound numerically: the methanol C─O stretching mode at 1030 cm^−1^, with a pump wavelength at 960 nm and Stokes wavelength at 1064 nm. The FWHM of the mode is reported to be 20 cm^−1^ (*25*). This corresponds to ω_p_ = 2.0 × 10^15^ rad s^−1^, ω_S_ = 1.8 × 10^15^ rad s^−1^, and Γ = 3.8 × 10^12^ rad s^−1^. Then, using Eq. 28, we can calculate

$$I_{\text{vacuum}}=\frac{{\hbar \omega }_{S}^{3}\Gamma }{2\pi c^{2}}=3.9\times 10^{6}\;W/m^{2}$$ (32)

For a tight-focusing Stokes beam operating at 100 mW, with 80-MHz repetition rate and 6-ps pulse width, we can calculate the peak intensity (note S3)

$$I_{\text{Stokes}}=\frac{P_{S}}{A_{S}\cdot \left(\tau \cdot f\right)}=6.1\times 10^{14}\;W/m^{2}$$ (33)

EF is then estimated to be EF = *I*_Stokes_/*I*_vacuum_ ≈ 2 × 10^8^. Remarkably, this number agrees well with the previous measurement of 3 × 10^8^ on this molecule (*13*), but is obtained from a theoretical point of view. The remaining minor discrepancy is likely due to the technical uncertainty in estimating several experimental parameters of microscopy. Overall, this is an important insight, as it reveals the origin and the extent of rate acceleration due to SRS.

In this context of stimulated Raman enhancement factor, it is instructive to discuss another commonly cited equation

$$\frac{r_{\text{SRS}}}{r_{\text{Raman}}}=n_{\text{Stokes}}+1$$ (34)

where 1 is the contribution from spontaneous contribution and *n*_Stokes_, denoting the number of photons in the Stokes mode, is the contribution from the external laser (*2*, *3*). The (*n* + 1) factor is a feature of bosons in general: The probability of a system emitting a boson into a quantum state already occupied by *n* bosons (of the same kind) is (*n* + 1) times larger than the probability of emission into that state if it is initially unoccupied. Despite its conceptual popularity in the SRS literature, Eq. 34 is rarely put to practical use due to the confusion of estimating *n*_Stokes_, the average number of Stokes photons in the optical mode (which is defined as electromagnetic standing waves within an optical resonator). Another complication is that Eq. 34 is referring to a particular mode, while the real experiments involve a large number of modes with different frequencies and orientations. A common myth is that *n*_Stokes_ is the number of photons per pulse, which happens to be around 10^8^.

We can actually recover Eq. 31 by using the photon number picture after including all modes. Let us consider a small rectangular cuboid region (a volume *V* defined by an area *A* and a length *L*) within which the pump laser and Stokes laser modes are defined. By definition, the laser intensity is equal to the number of photons contained in this region, *n*_Stokes_, multiplied by the energy per photon and divided by the cross-sectional area of the region and by the transit time through the region—that is

$$I_{\text{Stokes}}=\frac{n_{\text{Stokes}}\cdot \hbar \omega_{S}}{A\cdot \left(L/c\right)}=\frac{n_{\text{Stokes}}\cdot \hbar \omega_{S}}{V/c}$$ (35)

Then, *n*_Stokes_ can be related to light intensity via

$$n_{\text{Stokes}}=\frac{I_{\text{Stokes}}\cdot V}{\hbar \omega_{S}\cdot c}$$ (36)

We next consider the number of virtual photons from vacuum. As detailed previously (*14*), the number of modes of the electromagnetic field per unit frequency in volume *V* is given by $\rho_{\text{mode}}\left(\omega \right)=\frac{V\omega^{2}}{8\pi^{3}c^{3}}\mathrm{d\Omega}$. In QED, the ground state of vacuum consists of one virtual photon in each of these modes. Putting these together, this line of reasoning will suggest

$$\begin{array}{c} \text{EF}=\frac{n_{\text{Stokes}}}{1\cdot n_{\text{vac}\;\text{mode}}}=\frac{n_{\text{Stokes}}}{1\cdot \int \rho_{\text{mode}}\left(\omega \right)\cdot ∆\omega }=\frac{\frac{I_{\text{Stokes}}\cdot V}{\hbar \omega_{S}\cdot c}}{1\cdot \int \left(\frac{V\omega^{2}}{8\pi^{3}c^{3}}\mathrm{d\Omega}\right)\cdot \left(\frac{\pi \Gamma }{2}\right)}\\ =\frac{\frac{I_{\text{Stokes}}\cdot V}{\hbar \omega_{S}\cdot c}}{\frac{V\omega^{2}}{\pi^{2}c^{3}}\cdot \frac{\pi \Gamma }{2}}=\frac{I_{\text{Stokes}}}{I_{\text{vacuum}}} \end{array}$$ (37)

where volume *V* is canceled and the calculation of *n*_modes_ has evaluated the integration over the Raman linewidth (∆ω), all spatial angle (4π), and polarization (by a factor of 2). As expected, the final result recovers Eq. 31. Hence, one can generalize the photon number picture by including all modes and regard the EF as the ratio between *n*_Stokes_ and *n*_vacuum modes_, whose actual numbers both scale with the “imaginary” volume *V* under consideration.

### Signal to noise of SRS microscopy

The signal-to-noise ratio (SNR) is a critical property of any measurement technology. The quantitative capability of SRS allows us to predict the expected performance. In SRG, the relative gain experienced by the Stokes beam is

∆IStokesIStokes=PSRGAIStokes=NSRSA·(σRamanIvacuum)·Ip,SRS (38)

where *A* is the waist area of the laser focus. SRS measurements also need to overcome the noise of the detected laser beam, which is the Stokes beam in the SRG scheme. The number of Stokes photons collected during the τ_pixel,SRS_ period, *n*_Stokes_, can be expressed by

$$n_{\text{Stokes}}=\frac{I_{\text{Stokes}}}{\hbar \omega_{S}}\cdot A\cdot \tau_{\text{pulse}}\cdot f_{\text{rep}}\cdot \tau_{\text{pixel},\text{SRS}}$$ (39)

where *f*_rep_ is the repetition rate of the pulse train and τ_pulse_ is the pulse duration. The relative noise, after being normalized by the Stokes beam itself, is simply $\frac{\sqrt{n_{\text{Stokes}}}}{n_{\text{Stokes}}}$. SNR can be expressed as the ratio between the relative signal and the relative noise

$${\text{SNR}}_{\text{SRS}}=\frac{N_{\text{SRS}}}{A}\cdot \left(\frac{\sigma_{\text{Raman}}}{I_{\text{vacuum}}}\right)\cdot I_{p,\text{SRS}}\cdot \sqrt{n_{\text{Stokes}}}$$ (40)

**Fig. 2.**
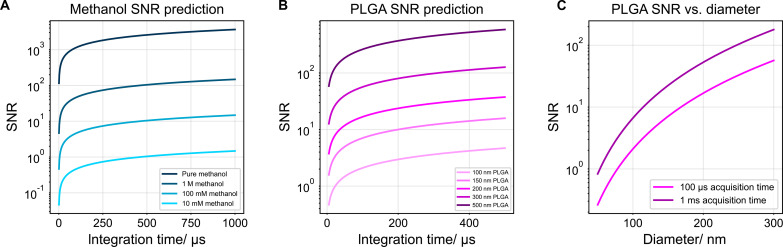
SNR prediction of SRS microscopy. (**A**) Estimation of SNR of methanol C─O stretching mode in solutions of different concentrations. (**B**) SNR estimation of PLGA nanoparticles observed by the C═O feature at 1768 cm^−1^. (**C**) Expected SNR versus the diameter of PLGA nanoparticles.

Plugging in the parameters from methanol measurement, we can plot the predicted SNR versus the integration time at different methanol concentrations (Fig. 2A and note S4).

SRS is finding applications in nanoparticle imaging (*26*–*32*). For nanoparticle-based measurements where the particle size is smaller than the focal volume, *n* is equal to the number of bonds contained in each individual particle. Assuming a spherical polymeric nanoparticle with a diameter *d*, a density of ρ, an average molecular weight of the repeating unit of MW, and each repeating unit has one target bond, we can estimate $N=\frac{1}{6}\pi d^{3}\cdot \frac{\rho }{\text{MW}}\cdot N_{A}$. For example, for poly(lactic-co-glycolic acid) (PLGA) nanoparticles observed at the C═O stretching mode at 1768 cm^−1^, we can obtain the SNR of nanoparticles of different sizes acquired at different time constants (note S4). Under 1-ms integration time, we can estimate that the theoretical detection limit would be ~50 nm for PLGA nanoparticles (Figure 2, B and C). Single-particle imaging of PLGA at ~100-nm level has indeed been recently demonstrated (*30*).

Equation 40 can also be used to calculate the pixel dwell time required to achieve reasonable SNR in high-speed chemical imaging experiments. For example, label-free imaging of cellular protein C─H mode has been one of the most important features of SRS. It is generally accepted that 20 to 30% of the cell volume is composed of proteins (200 to 300 g/liter). The average molecular weight for amino acids in proteins is considered to be around 100 Da, resulting in approximately 3 M effective amino acid concentration. Assuming that each amino acid on average has six C─H bonds, and plugging in the cross section for the entire C─H band in dimethyl sulfoxide (DMSO) (which also has six C─H bonds for every molecule) σ_Raman,C─H_ = 2.8 × 10^−29^ cm^2^ (*33*), we can estimate the SRS response from the cellular C─H signal assuming a 100 cm^−1^ linewidth. The results show that, to achieve SNR = 10 under 100-mW pump and Stokes power, a pixel dwell time of only ~35 ns is needed, equivalent to just a few pulses for an 80-MHz laser modulated at 50% duty cycle. This theoretical calculation explains the feasibility of video-rate cellular imaging in live cells, which was realized over a decade ago with a pixel dwell time of 100 ns (*34*–*36*).

### SRS microscopy is almost always more sensitive than spontaneous Raman microscopy for chemical imaging

Despite being well-established techniques on their own, which one of these two, spontaneous Raman microscopy or stimulated Raman microscopy, has a better detection limit for chemical imaging has not reached consensus in the Raman community. To some extent, the answer to this question underlies the fundamental importance of SRS microscopy. Experimentalists are drawing empirical conclusions from different sets of measurement conditions (including molecules, concentration, laser power, and acquisition time). While SNR comparison has been conducted from such experimental perspective (*37*), so far there has been no thorough discussion on the detection limit from a theoretical point of view of quantum mechanics. With various compounding experimental factors and without guidance from theory, fundamental insight can be elusive. Note that SRS was actually not used for sensitive detection during the majority part of its history. Between 1960s and the early 2000s, the applications of SRS had mostly been focused on the generation of coherent radiation and time-resolved spectroscopy (*38*–*43*), which cannot be done by spontaneous Raman scattering. Hence, this question of sensitivity comparison did not exist until relatively recently.

When SNR_SRS_ in Eq. 40 reaches 1, we can solve for the limit of detection ${\tilde{N}}_{\text{SRS}}$ as

$${\tilde{N}}_{\text{SRS}}=\frac{A\cdot I_{\text{vacuum}}\cdot \sqrt{n_{\text{Stokes}}}}{\sigma_{\text{Raman}}\cdot I_{p,\text{SRS}}\cdot n_{\text{Stokes}}}$$ (41)

Our strategy is to consider whether this lowest concentration can be lower than the corresponding limit of spontaneous Raman microscopy. If it does, it means that SRS microscopy can claim successful detection when spontaneous Raman microscopy is about to fail.

We need to define the fundamental detection limit of spontaneous Raman microscopy. For an acquisition time of τ_pixel,Raman_, the total energy of the Stokes signal is then

$$E_{\text{Raman}}=\eta \cdot N_{\text{Raman}}\cdot \sigma_{\text{Raman}}\cdot I_{p,\text{Raman}}\cdot \tau_{\text{pixel},\text{Raman}}$$ (42)

where η denotes the overall collection efficiency considering all contributions from the collection solid angle, objective, filters, confocal pinhole, gratings, and the camera. Note that, as the collection optics in SRS microscopy are much simpler (without the need for collection objective, confocal pinhole, or dispersive grating) and much more efficient (the SRS signal is directional unlike spontaneous Raman scattering), we assume 100% efficiency there. We argue that the criteria for successful detection in spontaneous Raman microscopy would be the detection of at least one photon in the detector. This means that $E_{\text{Raman}}=\hbar \omega_{S}$ in Eq. 42 defines the limit of detection by spontaneous Raman microscopy. Any energy below this level would be deemed impossible by the particle nature of light. Hence, we can solve for

$${\tilde{N}}_{\text{Raman}}=\frac{\hbar \omega_{S}\cdot A}{\eta \cdot \sigma_{\text{Raman}}\cdot P_{p,\text{Raman}}\cdot \tau_{\text{pixel},\text{Raman}}}$$ (43)

where *A* denotes the laser focus area and $P_{p,\text{Raman}}$ is the power of the incident pump laser beam. Equation 43 should be regarded as the best-case scenario, because it does not include any noise term. Real-world noise contributions from residual fluorescence background (which is nearly inevitable for biological imaging) and detector dark noise (which is also unavoidable given the detector technology) would further reduce the detection sensitivity of spontaneous Raman microscopy.

Let us consider whether ${\tilde{N}}_{\text{SRS}}$ dictated by Eq. 41 can be lower than ${\tilde{N}}_{\text{Raman}}$ dictated by Eq. 43. If it does, then we can prove that SRS exhibits superior detection sensitivity than spontaneous Raman microscopy. Substituting Eq. 39 for $n_{\text{Stokes}}$, Eq. 41 follows

$${\tilde{N}}_{\text{SRS}}=\frac{A\cdot I_{\text{vacuum}}\cdot \sqrt{n_{\text{Stokes}}}}{\sigma_{\text{Raman}}\cdot I_{p,\text{SRS}}\cdot \frac{I_{\text{Stokes}}}{\hbar \omega_{S}}\cdot A\cdot \tau_{\text{pulse}}\cdot f_{\text{rep}}\cdot \tau_{\text{pixel},\text{SRS}}}$$ (44)

The product of the parameters of $I_{p,\text{SRS}}\cdot A\cdot \tau_{\text{pulse}}\cdot f_{\text{rep}}$ is exactly the time-averaged power of the pump beam ${\bar{P}}_{p,\text{SRS}}$. For a fair comparison, we are using pump lasers with the same average power in both SRS and spontaneous Raman microscopy. Similarly, we shall also assign the same pixel dwell time for both. Hence, we assign ${\bar{P}}_{p,\text{SRS}}=P_{p,\text{Raman}}$ and $\tau_{\text{pixel},\text{SRS}}=\tau_{\text{pixel},\text{Raman}}$. With all these considerations, the ratio between Eq. 43 and Eq. 44 can be drastically simplified to

$$\frac{{\tilde{N}}_{\text{Raman}}}{{\tilde{N}}_{\text{SRS}}}=\frac{1}{\eta \cdot }\frac{I_{\text{Stokes}}}{I_{\text{vacuum}}\cdot \sqrt{n_{\text{Stokes}}}}$$ (45)

Since both the numerator and the denominator of Eq. 45 contain information on the Stokes beam, we attempt to express one by the other. It is appealing to convert the ratio between *I*_Stokes_ and *I*_vacuum_ to the ratio between photon numbers

$$\frac{I_{\text{Stokes}}}{I_{\text{vacuum}}}=\frac{\frac{I_{\text{Stokes}}}{\hbar \omega_{S}}\cdot A\cdot \tau_{\text{pulse}}\cdot f_{\text{rep}}\cdot \tau_{\text{pixel},\text{SRS}}}{\frac{I_{\text{vacuum}}}{\hbar \omega_{S}}\cdot A\cdot \tau_{\text{pulse}}\cdot f_{\text{rep}}\cdot \tau_{\text{pixel},\text{SRS}}}=\frac{n_{\text{Stokes}}}{n_{\text{vacuum}}}$$ (46)

where in the second step we have introduced a new quantity, *n*_vacuum_, as the number of effective virtual photon from the vacuum fluctuation. It can be regarded as an effective light source with a peak intensity of *I*_vacuum_ operating with the same spatial and temporal dynamics as the pump/Stokes beam in SRS. With the introduction of photon numbers, Eq. 45 then transforms to

$$\frac{{\tilde{N}}_{\text{Raman}}}{{\tilde{N}}_{\text{SRS}}}=\frac{1}{\eta \cdot }\frac{\sqrt{n_{\text{Stokes}}}}{n_{\text{vacuum}}}$$ (47)

which, in the ideal scenario where η = 100%, would reduce to a remarkably simple relation.

We can numerically evaluate Eq. 47 by adopting the experimental parameters in microscopy practice. Plugging the expression of *n*_Stokes_, *n*_vacuum_, and *I*_vacuum_, and defining the average power of the Stokes beam, it follows that

$$\frac{{\tilde{N}}_{\text{Raman}}}{{\tilde{N}}_{\text{SRS}}}=\frac{1}{\eta }\cdot \frac{\sqrt{\frac{{\bar{P}}_{\text{Stokes}}}{\hbar \omega_{S}\cdot \tau_{\text{pixel}}}}}{\frac{\omega_{S}^{2}\Gamma }{2\pi c^{2}}\cdot A\cdot \tau_{\text{pulse}}\cdot f_{\text{rep}}}$$ (48)

Plugging the typical SRS microscopy conditions we used in Eq. 33, the numerical evaluation yields

$$\frac{{\tilde{N}}_{\text{Raman}}}{{\tilde{N}}_{\text{SRS}}}=\frac{1}{\eta \cdot }\sqrt{\frac{0.08\;s}{\tau_{\text{pixel}}}}$$ (49)

Considering the various signal loss from the collection solid angle, objective, filters, confocal pinhole, gratings, and the camera, we take η = 10%, which is rather generous for common micro-Raman systems in the laboratory. Then, a reasonable pixel dwell time of 0.8 ms will result in $\frac{{\tilde{N}}_{\text{Raman}}}{{\tilde{N}}_{\text{SRS}}}=100$, indicating that SRS microscopy exhibits 100 times lower detection limit compared to spontaneous Raman microscopy.

The threshold value that would enable $\frac{{\tilde{N}}_{\text{Raman}}}{{\tilde{N}}_{\text{SRS}}}>1$ can be readily found

$$\tau_{\text{pixel}}<8\;s$$ (50)

The implication of this numerical result is immense. Eight seconds is substantially longer than the pixel dwell time (mostly less than 1 s) used in almost all spontaneous and stimulated Raman microscopy. Moreover, as elaborated above, real-world noise contributions from fluorescence background and detector noise would further worsen the sensitivity of spontaneous Raman microscopy. Hence, we can safely conclude that stimulated Raman microscopy is almost always more sensitive for any practical chemical imaging experiment on a single Raman mode.

### Vibrational population saturation

Vibrational saturation is typically not a concern for spontaneous Raman due to the slow rate of transition. SRS, on the other hand, is susceptible to saturation effects, particularly at high laser peak powers and/or for molecules with large cross sections. SRS saturation occurs when the rate of transition approaches the vibrational relaxation rate. Assuming the same SRS experiment condition as previously defined in the methanol measurement, then the rate of transition for a single molecule can be calculated based on Eq. 30 as *r*_SRS,C─O_ = 5.6 × 10^7^ s^−1^. This corresponds to an average lifetime of ~18 ns, which is much longer than the typical vibrational relaxation rate (~1 ps), so saturation is unlikely to occur. However, for a molecule with a much larger cross section, the rate of transition will be greatly enhanced. For example, Carbow 3-yne (σ_Raman_ = 2.2 × 10^−26^ cm^2^) excited under the same conditions will experience a rate *r*_SRS,sat_ = 2 × 10^12^ s^−1^, which is comparable to vibrational relaxation rate. For molecules under electronic resonance conditions, this saturation would occur at very low power. The saturated SRS process has found interesting utility in achieving super-resolution SRS imaging (*44*, *45*). The newly introduced formula shall allow us to directly calculate vibrational transition rates at different power levels, thereby predicting and optimizing SRS saturations in a quantitative manner.

**Fig. 3.**
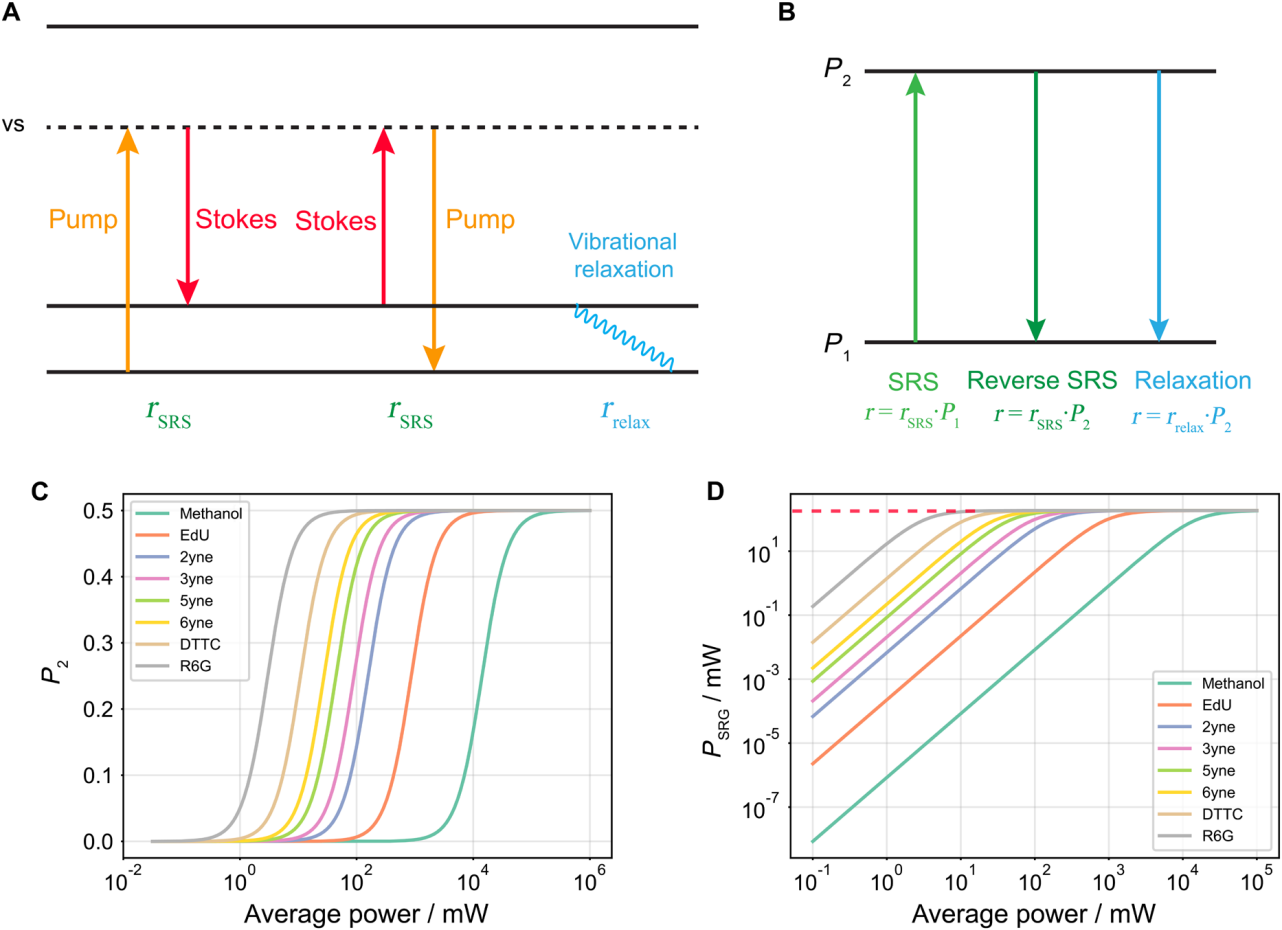
Prediction of SRS saturation. (**A**) Three competing processes are happening simultaneously in SRS experiments. First, a ground state molecule can absorb a pump photon and emit a Stokes photon back to the vibrational excited state. The reverse process is happening at the same rate for electrons starting from the vibrational excited state. Last, excited molecules can go through vibrational relaxation at a constant speed. (**B**) The three processes can be modeled using a two-state system. (**C**) Calculated *P*_2_, i.e., the fraction of molecules in the excited vibrational mode, at different power levels. (**D**) Expected SRG signal of different molecules under different pump and Stokes powers. Note that under the current theory, the SRG signal of all molecules converges to the scenario where transition is limited by vibrational relaxation, giving a power ceiling of approximately 0.2 W, which can be calculated by (*n*/2)·r_relax_·ℏω_S_. All calculations are assuming an effective laser spot area of approximately 3 × 10^−13^ m^2^ (note S3).

We can establish a simple two-state model (details in note S5) to describe this process quantitatively (Fig. 3, A and B). The corrected SRS signal after considering the saturation effect becomes

$$P_{\text{SRS},\text{corrected}}=\frac{N\cdot r_{\text{relax}}\cdot \hbar \omega_{S}\cdot P_{\text{SRG}}}{2P_{\text{SRG}}+N\cdot r_{\text{relax}}\cdot \hbar \omega_{S}}$$ (51a)

which clearly displays a saturation behavior. The corresponding rate equation can similarly be obtained

$$R_{\text{SRS},\text{corrected}}=N\cdot \frac{r_{\text{relax}}\cdot r_{\text{SRS}}}{2r_{\text{SRS}}+r_{\text{relax}}}$$ (51b)

When the effect is small, Eq. 51a recovers *P*_SRG_; when the effect is pronounced, it plateaus to (*N*/2)·*r*_relax_·ℏω_S_, which determines the maximum signal within each pulse.

To numerically evaluate this process, we use the vibrational lifetime [0.5 ps (*46*)] of methanol C─O stretching mode as a representative. We assume there are 10^6^ molecules inside the focal volume, which corresponds to approximately 8 mM in solution. The final SRS signal can be directly estimated from Eq. 51. For simplicity, we assume the same laser parameter used on methanol C─O measurement and ϕ_pump_ = ϕ_Stokes_. If we plug in the cross sections for common molecules and calculate the rates under different average laser powers (with 80-MHz repetition rate), we could readily predict SRS saturation (Fig. 3, C and D).

From Fig. 3 (C and D), we can see that small molecules such as methanol and 5-ethynyl-2′-deoxyuridine (EdU) maintain the linearity in most of the commonly used conditions in SRS experiments. This suggests that there is still much room to further improve the detection sensitivity via laser optimization (for example, higher peak power from lower repetition rates). Engineered conjugated systems such as Carbow, on the other hand, start to saturate at tens of milliwatts. Dyes experiencing electronic resonance saturate more easily at ~1 mW, which is consistent with previous experiment measurements (*47*). This predicted onset of saturation suggests their application in super-resolution SRS imaging. Note that our calculations are based on commercial lasers with 80-MHz repetition rate. Outcomes under different laser sources with varied repetition rates can be readily predicted. In reality, the actual saturation behavior may deviate from this prediction due to different molecular traits of relaxation and temporal dynamics of laser pulse. More sophisticated models can be built on the foundation of the rate equation (Eq. 51).

### Stimulated Raman photothermal microscopy

Very recently, stimulated Raman photothermal (SRP) microscopy was introduced, and its versatile applications on viral particles, cells, and tissues have been demonstrated (*48*). SRP explores the energy deposition of the SRS process, which pumps molecules to their vibrationally excited states. The subsequent relaxation heats up the surroundings and induces refractive index changes, which is detected as a thermal lens effect in SRP.

One crucial parameter is the amount of energy deposition at the laser focus, which is the source of the subsequent temperature elevation. Together with the SRS rate equation (Eq. 26), one can predict the amount of energy deposition after every pair of laser pulses as

$$E_{\text{thermal}}=R_{\text{SRG}}\cdot \tau_{\text{pulse}}\cdot \hbar \omega_{0}$$ (52)

where $\tau_{\text{pulse}}$ is the laser pulse width (in seconds) and $\hbar \omega_{0}$ is the energy of vibrational transition. Using the experimental parameters of 25 mW (modulated at 50% duty cycle) for the Stokes beam and 15 mW for the pump beams on sample at 80 MHz (6-ps pulse), on the broad C─H mode of DMSO that has a Raman cross section of 2.8 × 10^−29^ cm^2^ (with 100 cm^−1^ Raman linewidth) (*33*), Eq. 52 predicts the energy deposition to be about 7.9 fJ after each pair of laser pulses. This agrees well with 8.7 fJ extrapolated from SRS experiments (*48*).

## DISCUSSION

The theory underlying SRS science and technology appears to often lag behind the experiments. The discovery of SRS effect was serendipitous, as the experimental observation of Raman-related coherent radiation on nitrobenzene in 1962 was accidental and not predicted by theory ahead of time (*49*). The immediate applications in Raman-based shifter and Raman lasers around 1960s and 1970s were built on this property of light conversion (*38*), and the subsequent applications in time-resolved spectroscopy such as impulsive SRS (ISRS) and femtosecond SRS (FSRS) since the 1980s were leveraging the ultrafast nature of the excitation pulses (*40*, *41*, *43*). These two major directions of SRS do not have counterparts in spontaneous Raman scattering and do not involve sensitive detection. In this sense, SRS being a superior microscopy method for chemical imaging than spontaneous Raman microscopy, albeit believed empirically, was not rigorously predicted. Even more than a decade after its invention, the core question regarding the sensitivity comparison had remained challenging to address quantitatively. Now, the presented theory can predict the absolute signal of SRS microscopy from QED, and that SRS microscopy shall be almost always more sensitive than spontaneous Raman microscopy for chemical imaging. Thus, we hope that this study offers a theoretical framework for instrument design, experiment optimization, and performance analysis in the future, addressing an unmet need in the field of SRS microscopy.

## Supplementary Materials

### This PDF file includes:

Notes S1 to S5 Tables S1 and S2 References
