# Supplementary material for: Absolute signal of stimulated Raman scattering microscopy: A quantum electrodynamics treatment
Source: Sci Adv. 2024 Dec 11;10(50):eadm8424. doi: 10.1126/sciadv.adm8424 (PMC11633754; doi:10.1126/sciadv.adm8424)
Supplement: Supplementary file 1 — Notes S1 to S5 Tables S1 and S2 References [file sciadv.adm8424_sm.pdf]

Science Advances
AAAS

Supplementary Materials for

Absolute signal of stimulated Raman scattering microscopy: A quantum electrodynamics treatment

Wei Min and Xin Gao

Corresponding author: Wei Min, wm2256@columbia.edu

Sci. Adv. **10**, eadm8424 (2024)
DOI: 10.1126/sciadv.adm8424

This PDF file includes:

Notes S1 to S5
Tables S1 and S2
References

Note S1 Numerical estimation and comparison with experiments

It is insightful to relate the QED formulation of σ_{SRS} to a common but often mysterious dimensionless factor -- the fine structure constant α in fundamental physics:

$$\alpha = \frac{1}{4\pi\varepsilon_0}\frac{e^2}{\hbar c} \approx \frac{1}{137} \quad \text{(S1)}$$

To display the terms contained in the constant, we extract the elementary charge e and reduced Planck constant $\hbar$ from $\mathbb{M}$ in Eq. (16):

$$\sigma_{SRS} = \frac{\pi e^4 \omega_p \omega_S}{18\varepsilon_0^2 \hbar^2 c^2} \cdot \left| \sum_n \left\{ \frac{(\boldsymbol{D}^{fn} \cdot \boldsymbol{e}_S)(\boldsymbol{D}^{ni} \cdot \boldsymbol{e}_p)}{\omega_n - \omega_i - \omega_p - i\gamma} + \frac{(\boldsymbol{D}^{fn} \cdot \boldsymbol{e}_p)(\boldsymbol{D}^{ni} \cdot \boldsymbol{e}_S)}{\omega_n - \omega_i + \omega_S - i\gamma} \right\} \right|^2 \cdot G(\Omega_v) \quad \text{(S2)}$$

where the dipole moment $\boldsymbol{\mu}$ is reduced to $\boldsymbol{D}$, the displacement vector. Then it is easy to convert Eq. (S2) into

$$\sigma_{SRS} = \frac{8\pi^3}{9}\alpha^2 \cdot \omega_p \omega_S \cdot |\bar{\mathbb{M}}|^2 \cdot G(\Omega_v) \quad \text{(S3a)}$$

where we have re-defined the matrix element as

$$\bar{\mathbb{M}} = \sum_n \left\{ \frac{(\boldsymbol{D}^{fn} \cdot \boldsymbol{e}_S)(\boldsymbol{D}^{ni} \cdot \boldsymbol{e}_p)}{\omega_n - \omega_i - \omega_p - i\gamma} + \frac{(\boldsymbol{D}^{fn} \cdot \boldsymbol{e}_p)(\boldsymbol{D}^{ni} \cdot \boldsymbol{e}_S)}{\omega_n - \omega_i + \omega_S - i\gamma} \right\} \quad \text{(S3b)}$$

This compact form of Eq. (S3), together with the dimensionless nature of α, prompts us to readily verify the unit of σ_{SRS}. The numerator of $\bar{\mathbb{M}}$ apparently carries a dimension of length squared. The denominator of $\bar{\mathbb{M}}$ carries a dimension of ω^2, which cancels out with that of $\omega_p\omega_S$. The remaining $G(\Omega_v=\omega_p-\omega_S)$ exhibits a dimension of time (sec). Thus, the unit of the overall expression turns out to be $m^4 \cdot s$, as expected in Göppert-Mayer (1 GM = 10^{-50} $cm^4 \cdot s$), the unit introduced for two-photon absorption cross sections, σ_{TPA}(*22*). This dimensional analysis also sheds light on the physical meaning of the factors that determine σ_{SRS}: the $\frac{8\pi^3}{9}$ factor correlates to the polarization orientation and spatial angle, α^2 denotes the probability of a second-order field-matter interaction (as α governs the strength of electromagnetic interaction between charged particles and photons), $G(\Omega_v)$ captures the time scale, and $\omega_p\omega_S \cdot |\bar{\mathbb{M}}|^2$ together determines the spatial scale.

Numerical evaluation can be facilitated by Eq. (S3) expressed with the fine structure constant α. First we might be able to estimate the order of magnitude of σ_{SRS} for Raman modes in small molecules far away from electronic resonance. Strictly speaking, the exact evaluation requires a sum-over-state calculation, as the number of electronic states contributing to the polarizability is large. However, a back-of-the-envelope approximation is useful too. If we excite small molecules whole electronic state lies in the UV around 200 nm by a laser excitation around 1000 nm, then the angular frequency dependence in $\omega_p\omega_S \cdot |\bar{\mathbb{M}}|^2$ will produce a value close to $\left(\frac{1/1000}{1/200}\right)^2 \approx 0.04$

where the damping term can be neglected in far off resonance. We then assume the displacement D of the transition dipole moment to be 1/10 of the length of a bond (assumed to be around 1.5 Å) and the linewidth Γ of a typical Raman mode in condensed phase to be 15 cm^{-1}, equivalent to 4.5×10^{11} rad·s^{-1}. Then Eq. (S3) predicts

$$\sigma_{\mathrm{SRS}} \approx \frac{8\pi^3}{9} \times \left(\frac{1}{137}\right)^2 \times 0.04 \times (1.5 \times 10^{-11}\ \mathrm{m})^4 \times \frac{2}{\pi\times4.5\times10^{11}\ \mathrm{rad\cdot s^{-1}}} = 4 \times 10^{-2}\ \mathrm{GM} \qquad \text{(S4)}$$

This estimated result of 0.04 GM corresponds well with the experimentally measured value of 0.04 GM from C-O bond of small molecule methanol(*13*). While this is not meant to be a rigorous calculation, the agreement between theory and experiment is encouraging.

Electronic resonance can drastically enhance the cross sections of electronically coupled Raman modes, as the detuning approaches zero in the denominator. The summation over electronic states can be relaxed, provided the single resonant state has a large enough transition dipole moment. By applying the Born-Oppenheimer approximation of separability of electronic and vibrational wavefunction, resonance Raman has been shown to be dominated by the so-called Albrecht's A-term for strongly allowed electronic transitions and substantial nonorthogonality of Frank-Codon overlap factor(*17*, *50*). Let's assume both the laser excitation wavelength and the chromophore electronic absorption to be around 700 nm (i.e., exact electronic resonance) which is about 14,000 cm^{-1}, and the electronic linewidth (damping term) to be around 700 cm^{-1}, the frequency dependence of $\omega_\mathrm{p}\omega_\mathrm{S} \cdot |\overline{\mathbb{M}}|^2$ will produce $\left(\frac{14000\ \mathrm{cm}^{-1}}{700\ \mathrm{cm}^{-1}}\right)^2 = 400$. Note this factor is 10,000 folds higher than the far-off resonance case above. Electronic resonance also creates strong displacement D for electronically coupled Raman modes. For example, electronic transition dipole moments have been reported as 7.1 Debye (about 1.4 e·Å) for PM546 dye and 8.1 Debye (about 1.6 e·Å) for Rhodamine 123, respectively(*51*). If we take the transition displacement D as 1.6Å, the numerator of $|\overline{\mathbb{M}}|^2$ will produce another factor of 13,000 compared to the small molecule above. Finally we need to consider Frank-Codon overlap when evaluating the dipole moment elements of vibronic transitions. For strongly coupled transition, this overlap can be substantial according to Albrecht's theory if there is sizable shift of the excited state potential along the vibrational coordinate(*17*, *50*). Without loss of generality, it is assumed to be 0.1 here. Together, Eq. (S3) predicts the electronic resonant result to be

$$\sigma_{\mathrm{SRS}} \approx \frac{8\pi^3}{9} \times \left(\frac{1}{137}\right)^2 \times 400 \times (1.6 \times 10^{-10}\ \mathrm{m})^4 \times \frac{2}{\pi\times4.5\times10^{11}\ \mathrm{rad\cdot s^{-1}}} \times 0.1 \approx 5 \times 10^5\ \mathrm{GM} \qquad \text{(S5)}$$

Experimentally, in the exact electronic resource condition, R6G has a stimulated Raman cross section of 860,000 GM for the electronic coupled ring mode, and, similarly, IR820 chromophore has around 430,000 GM(*13*). Again, the agreement is satisfactory considering the crude approximation. The success of both the off-resonance case in Eq. (S4) and the electronic resonance case in Eq. (S5) indicates that the theory presented here can predict the outcome of SRS experiments from first principles.

Note S2 Rederivation in the form of pump loss

We start from Eq. (26):

$$R_{\text{SRS}} = N \cdot \sigma_{\text{SRS}}(\Omega) \cdot \phi_{\text{pump}} \cdot \phi_{\text{Stokes}} \tag{S6}$$

In the detection scheme of pump loss instead of Stokes gain, we can multiply $\hbar\omega_{\text{p}}$, the energy of the pump photons on both sides.

$$P_{\text{SRL}} = N \cdot \left(\frac{\sigma_{\text{Raman}}}{\phi_{\text{vacuum}}}\right) \cdot I_{\text{pump}} \cdot \phi_{\text{Stokes}} \tag{S7}$$

where P_{SRL} is the energy flux (J·s^{-1}) of the SRS signal in terms of SRL. We then multiply $\hbar\omega_{\text{S}}$ to both ϕ_{Stokes} and ϕ_{vacuum} of the first term in Eq. (16), we arrive at:

$$P_{\text{SRG}} = N \cdot \sigma_{\text{Raman}} \cdot I_{\text{pump}} \cdot \left(\frac{I_{\text{Stokes}}}{\phi_{\text{vacuum}} \cdot \hbar\omega_S}\right) \tag{S8}$$

Similarly, we can define

$$\phi_{\text{vacuum}} \cdot \hbar\omega_S = \frac{\omega_{\text{S}}^4 \Gamma}{2\pi c^2 \omega_{\text{p}}} \equiv I'_{\text{vacuum}} \tag{S9}$$

Finally, we have derived the following equation for absolute SRS signal from regular Raman cross section:

$$P_{\text{SRL}} = N \cdot \sigma_{\text{Raman}} \cdot I_{\text{pump}} \cdot \left(\frac{I_{\text{Stokes}}}{I'_{\text{vacuum}}} + \frac{1}{2}\frac{\hbar\omega_{\text{p}}}{\hbar\omega_{\text{S}}}\right) \tag{S10}$$

Since I_{vacuum} and I'_{vacuum} are connected by

$$\frac{I'_{\text{vacuum}}}{\hbar\omega_{\text{S}}} = \frac{I_{\text{vacuum}}}{\hbar\omega_{\text{p}}} \tag{S11}$$

we can also get

$$P_{\text{SRL}} = N \cdot \frac{\hbar\omega_{\text{p}}}{\hbar\omega_{\text{S}}} \cdot \sigma_{\text{Raman}} \cdot I_{\text{pump}} \cdot \frac{I_{\text{Stokes}}}{I_{\text{vacuum}}} \tag{S12}$$

And the rate form of the equation

$$R_{\text{SRL}} = N \cdot \sigma_{\text{Raman}} \cdot \phi_{\text{pump}} \cdot \frac{\phi_{\text{Stokes}}}{\phi_{\text{vacuum}}} \tag{S13}$$

And $R_{\text{SRL}} = R_{\text{SRG}}$, which is the expected result.

Note S3 laser parameters

The focal volume and the laser waist area of the SRS microscope were calculated based on the 1/e width ω as follows(*52*):

$$\omega_{xy} = \frac{0.325\lambda}{\sqrt{2}NA^{0.91}}\ (NA > 0.7) \quad \text{(S14)}$$

$$\omega_{z} = \frac{0.532\lambda}{\sqrt{2}} \frac{1}{n-\sqrt{(n^2-NA^2)}} \quad \text{(S15)}$$

Where *NA*=1.05 is the numeric aperture of the objective lens, n=1.518 is the refractive index of the immersive oil. Focal volume V_{TPE} and laser waist area A are then calculated as:

$$V_{TPE} = \pi^{\frac{3}{2}} \omega_{xy}^2 \omega_z \quad \text{(S16)}$$

$$A = \pi\left(\sqrt{2}\omega_{xy}\right)^2 \quad \text{(S17)}$$

Where $\sqrt{2}\omega_{xy}$ is the $1/e^2$ width of the laser spot. The parameters for methanol 1030 cm^{-1} mode are summarized as follows.

Assuming we are observing the methanol C-O bond at 1030 cm^{-1} with 100 mW pump and Stokes beam at 960 nm and 1064 nm, both operating at 80 MHz with a pulse duration of 6 ps. Effectively, this leads to a duty cycle of 4.8×10^{-4}. Assuming the lasers are tightly focused into the same spot, we can use the nonlinear focal spot relation mentioned above to calculate microscope parameters:

Laser source	Wavelength / nm	ω_{xy} / nm	ω_z / nm	V_{TPE} / aL	A/ m^2
Pump	960	211	856	212	2.80E-13
Stokes	1064	234	949	289	3.44E-13

V_{TPE} for pump laser is used as the final focal volume. For DMSO C-H band measurements at 2913cm^{-1}, the conditions are summarized as follows:

Laser source	Wavelength / nm	ω_{xy} / nm	ω_z / nm	V_{TPE} / aL	A/ m^2
Pump	810	178	723	128	2.00E-13
Stokes	1064	234	949	289	3.44E-13

Note S4 Calculation of SNR in SRS experiments

From Eq. (40), if we assume a solution measurement where $n_{\text{SRS}} = N_A \cdot [c] \cdot V$:

$$SNR = (N_{\text{A}} \cdot [c] \cdot V) \cdot \left(\sigma_{\text{Raman}} \cdot \frac{\phi_{\text{pump,peak}}}{\phi_{\text{vacuum}}}\right) \cdot \sqrt{\frac{\phi_{\text{Stokes,ave}}}{A} \cdot \tau} \qquad \text{(S18)}$$

Where τ is the integration time, A is the area of the Stokes beam. $A \cdot l$ has been replaced by V, which is the focal volume in the case of solution measurements. The average Stokes rate is used to model the actual read-out within the dwell time. Note that $N_{\text{A}} \cdot [c] \cdot V$ is just the number of molecules N. For measurement on non-solution-based targets such as nanoparticles, the generalized form on N still holds:

$$SNR = N \cdot \left(\sigma_{\text{Raman}} \cdot \frac{\phi_{\text{pump,peak}}}{\phi_{\text{vacuum}}}\right) \cdot \sqrt{\frac{\phi_{\text{Stokes,ave}}}{A} \cdot \tau} \qquad \text{(S19)}$$

Plug in the expression for ϕ_{vacuum}:

$$SNR = \left(\frac{2\pi c^2 \omega_{\text{p}}}{\omega_{\text{S}}^3 \Gamma}\right) \cdot N \cdot \sigma_{\text{Raman}} \cdot \phi_{\text{pump,peak}} \cdot \sqrt{\phi_{\text{Stokes,ave}}} \cdot \sqrt{\frac{\tau}{A}} \qquad \text{(S20)}$$

Which means SNR is proportional to the square root of Stokes power and linear to the pump power in the Stokes gain detection scheme. More specifically for solution, we have

$$SNR = \left(\frac{2\pi c^2 \omega_{\text{p}}}{\omega_{\text{S}}^3 \Gamma}\right) \cdot N_{\text{A}} \cdot V \cdot \sigma_{\text{Raman}} \cdot \phi_{\text{pump,peak}} \cdot \sqrt{\frac{\phi_{\text{Stokes,ave}}}{A}} \cdot [c] \cdot \sqrt{\tau} \qquad \text{(S21)}$$

The effective SRS focal volume for methanol C-O mode measurement is about 212 aL. For pure methanol at 24.7 M concentration, this corresponds to 3.2×10^9 molecules inside the focal volume. The spontaneous Raman cross section for single C-O stretching is reported at 9.0×10^{-31} cm^2. Plugging in all the experimental parameters, we have the numerical form for methanol:

$$SNR = 4.67 \times [c] \cdot \sqrt{\tau} \qquad \text{(S22)}$$

Where all units are expressed in SI units.

For the measurement on nanoparticles, because the focal volume is only partially occupied, n can be directly estimated from the size, density and molecular weight of the nanoparticle.

$$N = \frac{1}{6}\pi d^3 \cdot \frac{\rho}{M} \cdot N_{\text{A}} \qquad \text{(S23)}$$

Then we have:

$$SNR = \left(\frac{2\pi c^2 \omega_{\text{p}}}{\omega_{\text{S}}^3 \Gamma}\right) \cdot \frac{1}{6}\pi \cdot \frac{\rho}{M} \cdot N_{\text{A}} \cdot \sigma_{\text{Raman}} \cdot \phi_{\text{pump,peak}} \cdot \sqrt{\frac{\phi_{\text{Stokes,ave}}}{A}} \cdot d^3 \cdot \sqrt{\tau} \qquad \text{(S24)}$$

For example, the ester bond in PLGA, one of the most important FDA-approved carriers for nanomedicine, is measured to have a cross-section of 1.4×10^{-30} cm^2, with a bandwidth at ~40 cm^{-1}. The density of PLGA is estimated to be about 1.3 kg/m^3, and with an average molecular weight of 65 g/mol. Plugging in all the numbers, we have

$$SNR = 2.1 \times 10^{23} \sqrt{\tau} \cdot d^3 \qquad \text{(S25)}$$

Similarly, all numbers are in SI units.

Note S5 Vibrational population saturation two-state model

We can establish a simple two-state model to describe this process more quantitatively. In a typical SRS experiment, there are three competing processes (**Figure 3A**): The first is when a ground state vibration transits to vibrational excited state via absorbing a pump photon and emitting a Stokes photon; the second is the vibrational excited state returns to ground state through vibrational relaxation; the third is the vibrational excited state transits the ground state via absorbing a Stokes photon and emitting a pump photon. By virtue of detailed balancing, the first and the third process should share the same rate for each individual molecule, r_{SRS}. Vibrational relaxation rate r_{relax} is the inverse of the vibrational lifetime (typically ~ 0.1-10 ps(*53*)). Assume the probability of the molecule in the ground and excited states are P_1 and P_2, respectively. Obviously $P_1+P_2=1$, as shown in **Figure 3B**.The differential rate equations can be expressed as:

$$\frac{dP_1}{dt} = -r_{SRS} \cdot P_1 + r_{SRS} \cdot P_2 + r_{relax} \cdot P_2 \quad \text{(S26a)}$$

$$\frac{dP_2}{dt} = r_{SRS} \cdot P_1 - r_{SRS} \cdot P_2 - r_{relax} \cdot P_2 \quad \text{(S26b)}$$

In equilibrium state, $\frac{dP_1}{dt} = \frac{dP_2}{dt} = 0$. Then, we can get

$$P_1 = \frac{r_{SRS}+r_{relax}}{2r_{SRS}+r_{relax}} \quad \text{(S27a)}$$

$$P_2 = \frac{r_{SRS}}{2r_{SRS}+r_{relax}} \quad \text{(S27b)}$$

It is obvious when $r_{SRS} \gg r_{relax}$, P_1 and P_2 approaches ½. The difference

$$(P_1 - P_2) = \frac{r_{relax}}{2r_{SRS}+r_{relax}} = \frac{N \cdot r_{relax} \cdot \hbar\omega_S}{2P_{SRG}+N \cdot r_{relax} \cdot \hbar\omega_S} \quad \text{(S28)}$$

This population difference starts to diminish when the SRS rate is surpassing the r_{relax}. Then the corrected SRS signal after considering the saturation effect becomes

$$P_{SRS,corrected} = (P_1 - P_2) \cdot P_{SRG} = \frac{N \cdot r_{relax} \cdot \hbar\omega_S \cdot P_{SRG}}{2P_{SRG}+N \cdot r_{relax} \cdot \hbar\omega_S} \quad \text{(S29a)}$$

which clearly displays a saturation behavior. The corresponding rate equation can similarly be obtained:

$$R_{SRS,corrected} = (P_1 - P_2) \cdot R_{SRS} = N \cdot \frac{r_{relax} \cdot r_{SRS}}{2r_{SRS}+r_{relax}} \quad \text{(S29b)}$$

When the effect is small, Eq. (S29) recovers S_{SRG}; when the effect is pronounced, it plateaus to $(nN/2) \cdot r_{relax} \cdot \hbar\omega_S$, which determines the maximum signal within each pulse.

Table S1 Spontaneous Raman cross sections used in the text

molecule	Raman mode / cm^{-1}	pump wavelength / nm	cross sections(*13*) / cm^2
methanol	C-O (1030)	960	9.0E-31
DMSO(*33*)	C-H (2913)	810	2.8E-29
EdU	2125	868	2.4E-28
2yne	2226	860	7.2E-27
3yne	2183	863	2.2E-26
4yne	2141	866	4.8E-26
5yne	2100	870	9.2E-26
6yne	2066	872	2.4E-25
DTTC	1281	785	1.5E-24
R6G	1647	555	2.0E-23
IR820 (pre-resonance)	1628	907	5.8E-25

All differential cross sections are converted to total cross section using the formula provided in literature(*54*):

$$\sigma_R = \frac{8\pi}{3}\frac{1+2\rho}{1+\rho}\frac{\partial\sigma}{\partial\Omega_{\parallel\perp}} \quad \text{(S30)}$$

Where σ_R is the total cross section, and ρ is the depolarization ratio measured/calculated for the corresponding band(*33*). Note that here anisotropic Raman scattering is assumed to be consistent with real molecules.

Table S2 units of stated physical properties in the text

Type	Name	Symbol	common unit
Laser parameters	power	P	W
	number of photons	n	1
	laser spot area	A	cm^2
	pulse duration	τ	ps
	rep rate	f	MHz
	photon flux	ϕ	$photon \cdot cm^{-2} \cdot s^{-1}$
	angular frequency	ω	$rad \cdot s^{-1}$
	spectral photon flux	$F(\omega)$	$photon \cdot s^{-1} \cdot cm^{-2} \cdot rad^{-1} \cdot s$
	Light intensity	I	W/m^2
Raman experiments	SRS signal (power)	P	W
	Number of molecules/bonds	N	1
	Number of molecules under the detection limit	$\widetilde{N}$	1
	rate (single bond)	r	s^{-1}
	rate (collective behavior)	R	s^{-1}
Sample info	SRS cross section	σ_{SRS}	$1\ GM = 10^{-50}\ cm^4 \cdot s \cdot photon^{-1}$
	attenuation coefficient	ε	$L \cdot mol^{-1} \cdot cm^{-1}$
	concentration	$[c]$	mol/L
	number of molecules	N	1
	length	l	cm

REFERENCES AND NOTES

1. J.-X. Cheng, W. Min, Y. Ozeki, D. Polli, *Stimulated Raman Scattering Microscopy: Techniques and Applications* (Elsevier, 2021).

2. W. Min, C. W. Freudiger, S. Lu, X. S. Xie, Coherent nonlinear optical imaging: Beyond fluorescence microscopy. *Annu. Rev. Phys. Chem.* **62**, 507–530 (2011).

3. R. C. Prince, R. R. Frontiera, E. O. Potma, Stimulated Raman scattering: From bulk to nano. *Chem. Rev.* **117**, 5070–5094 (2017).

4. H. Rigneault, P. Berto, Tutorial: Coherent Raman light matter interaction processes. *APL Photonics* **3**, 091101 (2018).

5. J. Huang, M. Ji, Stimulated Raman scattering microscopy on biological cellular machinery. *J. Innov. Opt. Health Sci.* **16**, 2230010 (2023).

6. Y. Ozeki, F. Dake, S. Kajiyama, K. Fukui, K. Itoh, Analysis and experimental assessment of the sensitivity of stimulated Raman scattering microscopy. *Opt. Express* **17**, 3651–3658 (2009).

7. D. Zhang, M. N. Slipchenko, J.-X. Cheng, Highly sensitive vibrational imaging by femtosecond pulse stimulated Raman loss. *J. Phys. Chem. Lett.* **2**, 1248–1253 (2011).

8. P. Nandakumar, A. Kovalev, A. Volkmer, Vibrational imaging based on stimulated Raman scattering microscopy. *New J. Phys.* **11**, 033026 (2009).

9. L. Shi, R. R. Alfano, *Deep Imaging in Tissue and Biomedical Materials: Using Linear and Nonlinear Optical Methods* (CRC Press, 2017).

10. C. W. Freudiger, W. Min, B. G. Saar, S. Lu, G. R. Holtom, C. He, J. C. Tsai, J. X. Kang, X. S. Xie, Label-free biomedical imaging with high sensitivity by stimulated Raman scattering microscopy. *Science* **322**, 1857–1861 (2008).

11. F. Hu, L. Shi, W. Min, Biological imaging of chemical bonds by stimulated Raman scattering microscopy. *Nat. Methods* **16**, 830–842 (2019).

12. B. Manifold, D. Fu, Quantitative stimulated Raman scattering microscopy: Promises and pitfalls. *Annu. Rev. Anal. Chem.* **15**, 269–289 (2022).

13. X. Gao, X. Li, W. Min, Absolute stimulated Raman cross sections of molecules. *J. Phys. Chem. Lett.* **14**, 5701–5708 (2023).

14. W. Min, X. Gao, Raman scattering and vacuum fluctuation: An Einstein-coefficient-like equation for Raman cross sections. *J. Chem. Phys.* **159**, 194103 (2023).

15. W. Min, X. Gao, Quantum mechanical treatment of stimulated Raman cross sections. arXiv:2312.07473 [physics.chem-ph] (2023).

16. D. P. Craig, T. Thirunamachandran, *Molecular Quantum Electrodynamics: An Introduction to Radiation-Molecule Interactions* (Courier Corporation, 1998).

17. R. Loudon, *The Quantum Theory of Light* (OUP Oxford, 2000).

18. D. A. Long, *The Raman Effect: A Unified Treatment of the Theory of Raman Scattering by Molecules* (Wiley, 2002).

19. G. C. Schatz, M. A. Ratner, *Quantum Mechanics in Chemistry* (Dover Publications, 2002).

20. Y. R. Shen, *The Principles of Nonlinear Optics* (Wiley, 2002).

21. P. W. Milonni, *The Quantum Vacuum: An Introduction to Quantum Electrodynamics* (Academic Press, 2013).

22. R. W. Boyd, *Nonlinear Optics, Fourth Edition* (Academic Press, 2020).

23. A. Einstein, The quantum theory of radiation. *Phys. Z.* **18**, 121 (1917).

24. L. Wei, W. Min, Electronic preresonance stimulated Raman scattering microscopy. *J. Phys. Chem. Lett.* **9**, 4294–4301 (2018).

25. K. Tanabe, S. Tsuzuki, Raman linewidth study of intermolecular interactions of methanol in aqueous solution. *Spectrochim. Acta A Mol. Spectrosc.* **42**, 611–614 (1986).

26. X. Lang, X. Gao, M. Wei, N. Qian, W. Min, Bioorthogonal chemical imaging of solid lipid nanoparticles with minimal labeling by stimulated Raman scattering microscopy. *Nat. Sci.* **3**, e202103304 (2023).

27. J. Ao, G. Xu, H. Wu, L. Xie, J. Liu, K. Gong, X. Ruan, J. Han, K. Li, W. Wang, T. Chen, M. Ji, L. Zhang, Fast detection and 3D imaging of nanoplastics and microplastics by stimulated Raman scattering microscopy. *Cell Rep. Phys. Sci.* **4**, 101623 (2023).

28. L. Shi, H. Jang, Perspectives on SRS imaging of nanoparticles. *Acc. Mater. Res.* **4**, 726–728 (2023).

29. S. Vanden-Hehir, S. A. Cairns, M. Lee, L. Zoupi, M. P. Shaver, V. G. Brunton, A. Williams, A. N. Hulme, Alkyne-tagged PLGA allows direct visualization of nanoparticles in vitro and ex vivo by stimulated Raman scattering microscopy. *Biomacromolecules* **20**, 4008–4014 (2019).

30. X. Gao, X. Lang, E. El Khoury, M. Wei, N. Qian, W. Min, Quantitative label-free chemical imaging of PLGA nanoparticles in cells and tissues with single-particle sensitivity. *Nano Lett.* **24**, 1024–1033 (2024).

31. N. Qian, X. Gao, X. Lang, H. Deng, T. M. Bratu, Q. Chen, P. Stapleton, B. Yan, W. Min, Rapid single-particle chemical imaging of nanoplastics by SRS microscopy. *Proc. Natl. Acad. Sci. U.S.A.* **121**, e2300582121 (2024).

32. M. Wei, N. Qian, X. Gao, X. Lang, D. Song, W. Min, Single-particle imaging of nanomedicine entering the brain. *Proc. Natl. Acad. Sci. U.S.A.* **121**, e2309811121 (2024).

33. K. H. Burns, P. Srivastava, C. G. Elles, Absolute cross sections of liquids from broadband stimulated Raman scattering with femtosecond and picosecond pulses. *Anal. Chem.* **92**, 10686–10692 (2020).

34. B. G. Saar, C. W. Freudiger, C. M. Stanley, G. R. Holtom, X. S. Xie, Video-rate molecular imaging in vivo with stimulated Raman scattering. *Science* **330**, 1368–1370 (2010).

35. J. L. Suhalim, J. C. Boik, B. J. Tromberg, E. O. Potma, The need for speed. *J. Biophotonics* **5**, 387–395 (2012).

36. Y. Ozeki, W. Umemura, Y. Otsuka, S. Satoh, H. Hashimoto, K. Sumimura, N. Nishizawa, K. Fukui, K. Itoh, High-speed molecular spectral imaging of tissue with stimulated Raman scattering. *Nat. Photon* **6**, 845–851 (2012).

37. H. Rigneault, Y. Ozeki, "Chapter 2—Sensitivity and noise in SRS microscopy" in *Stimulated Raman Scattering Microscopy*, J.-X. Cheng, W. Min, Y. Ozeki, D. Polli, Eds. (Elsevier, 2022), pp. 21–40.

38. G. Eckhardt, D. P. Bortfeld, M. Geller, Stimulated emission of Stokes and anti-Stokes Raman lines from diamond, calcite, and α-sulfur single crystals. *Appl. Phys. Lett.* **3**, 137–138 (1963).

39. D. von der Linde, A. Laubereau, W. Kaiser, Molecular vibrations in liquids: Direct measurement of the molecular dephasing time; determination of the shape of picosecond light pulses. *Phys. Rev. Lett.* **26**, 954–957 (1971).

40. A. Laubereau, W. Kaiser, Vibrational dynamics of liquids and solids investigated by picosecond light pulses. *Rev. Mod. Phys.* **50**, 607–665 (1978).

41. L. Dhar, J. A. Rogers, K. A. Nelson, Time-resolved vibrational spectroscopy in the impulsive limit. *Chem. Rev.* **94**, 157–193 (1994).

42. M. Yoshizawa, M. Kurosawa, Femtosecond time-resolved Raman spectroscopy using stimulated Raman scattering. *Phys. Rev. A* **61**, 013808 (1999).

43. P. Kukura, D. W. McCamant, R. A. Mathies, Femtosecond stimulated Raman spectroscopy. *Annu. Rev. Phys. Chem.* **58**, 461–488 (2007).

44. L. Gong, H. Wang, Breaking the diffraction limit by saturation in stimulated-Raman-scattering microscopy: A theoretical study. *Phys. Rev. A* **90**, 013818 (2014).

45. L. Gong, W. Zheng, Y. Ma, Z. Huang, Saturated stimulated-Raman-scattering microscopy for far-field superresolution vibrational imaging. *Phys. Rev. Appl.* **11**, 034041 (2019).

46. S. Meiselman, O. Cohen, M. F. DeCamp, V. O. Lorenz, Measuring vibrational coherence lifetimes in liquid methanol using transient coherent Raman scattering. *J. Phys. Conf. Ser.* **497**, 012004 (2014).

47. L. Shi, H. Xiong, Y. Shen, R. Long, L. Wei, W. Min, Electronic resonant stimulated Raman scattering micro-spectroscopy. *J. Phys. Chem. B* **122**, 9218–9224 (2018).

48. Y. Zhu, X. Ge, H. Ni, J. Yin, H. Lin, L. Wang, Y. Tan, C. V. Prabhu Dessai, Y. Li, X. Teng, J.-X. Cheng, Stimulated Raman photothermal microscopy toward ultrasensitive chemical imaging. *Sci. Adv.* **9**, eadi2181 (2023).

49. G. Eckhardt, R. W. Hellwarth, F. J. McClung, S. E. Schwarz, D. Weiner, E. J. Woodbury, Stimulated Raman scattering from organic liquids. *Phys. Rev. Lett.* **9**, 455–457 (1962).

50. A. C. Albrecht, On the theory of Raman intensities. *J. Chem. Phys.* **34**, 1476–1484 (1961).

51. P.-H. Chung, C. Tregidgo, K. Suhling, Determining a fluorophore's transition dipole moment from fluorescence lifetime measurements in solvents of varying refractive index. *Methods Appl. Fluoresc.* **4**, 045001 (2016).

52. W. R. Zipfel, R. M. Williams, W. W. Webb, Nonlinear magic: Multiphoton microscopy in the biosciences. *Nat. Biotechnol.* **21**, 1369–1377 (2003).

53. D. W. Oxtoby, Vibrational relaxation in liquids. *Annu. Rev. Phys. Chem.* **32**, 77–101 (1981).

54. W. R. Silva, E. L. Keller, R. R. Frontiera, Determination of resonance Raman cross-sections for use in biological SERS sensing with femtosecond stimulated Raman spectroscopy. *Anal. Chem.* **86**, 7782–7787 (2014).
